# Exposure to Mixtures of Pollutants in Mexican Children from Marginalized Urban Areas

**DOI:** 10.29024/aogh.912

**Published:** 2018-07-27

**Authors:** Rogelio Flores-Ramírez, Francisco J. Pérez-Vázquez, Susanna E. Medellín-Garibay, Andrea Camacho Aldrete, Moisés Roberto Vallejo-Pérez, Lorena Díaz de León-Martínez, Leticia Carrizales Yáñez, Fernando Díaz-Barriga

**Affiliations:** 1 Coordinación para la Innovación y Aplicación de la Ciencia y la Tecnología (CIACYT), Universidad Autónoma de San Luis Potosí, Avenida Sierra Leona No. 550, CP 78210, Colonia Lomas Segunda Sección, San Luis Potosí, SLP, MX; 2 Centro de Investigación Aplicada en Ambiente y Salud, CIACYT, Facultad de Medicina, Universidad Autónoma de San Luis Potosí. Av. Venustiano Carranza 2405, CP 78210, San Luis Potosí, SLP, MX

## Abstract

**Background:**

Exposure to contaminant mixtures in developing countries is an important public health issue. Children are identified as the most susceptible group to adverse health effects due to the exposure.

**Objective:**

The aim of this study was to conduct a screening for mixture pollutants in Mexican children in urban marginalized communities.

**Methods:**

We analyzed children (aged 6–12 years old) who resided in four urban marginalized communities in San Luis Potosi, Mexico: i) Bellas Lomas (BEL), a site with vehicular traffic; ii) Tercera Chica (TC), a site with brick kilns; Iii) Rincon de San Jose (SJR), a site with a hazardous waste landfill; and (iv) Morales (MOR) a metallurgical zone with copper-arsenic and electrolytic zinc smelters. Polycyclic Aromatic Hydrocarbons (1-hydroxypyrene (1-OHP)), benzene (trans, trans-muconic acid (t,t-MA), manganese, arsenic and fluoride were quantified in urine and lead in blood samples.

**Findings:**

Our results indicate that median exposures to manganese were 4.4, 5.2, 5.8 and 6.3 µg/L for BEL, TC, SJR and MOR, respectively. For BEL, fluoride was present at a higher concentration with 2.3 mg/L followed by MOR, TC and SJR with 1.7, 1.5 and 1.2 mg/L respectively. The highest concentrations of arsenic that were found were 11 µg/L in MOR and lead concentration was reported between 4.2 and 6.8 µg/dL, in BEL, TC and MOR. 1-OHP and t,t-MA were higher in TC (0.23 µmol/mol creatinine (cr), 429.7 µg/g cr, respectively) followed by SJR (0.09 µmol/mol cr, 427.4 µg/g cr), MOR (0.03 µmol/mol cr, 258.6 µg/g cr) and BEL (0.06 µmol/mol cr, 220.6 µg/g cr).

**Conclusion:**

Considering the large number of people, especially children, exposed to multiple pollutants, it is important to design effective intervention programs that reduce exposure and the resultant risk in the numerous urban marginalized communities in Mexico.

## Introduction

Population growth in cities is a global and progressive phenomenon. In 2008, the Organization of United Nations reported that more than half of the human population lives in urban areas [1]. In large cities, the proliferation of neighborhoods of precarious habitat is becoming more frequent [2,3]. The consequences of population growth and urbanization without planning and control have widened the social gap within cities and led to significant poverty belts with lack of employment, housing, security and protection of the environment [4,5]. For example, in Mexico at 2010, approximately 86 million people lived in urban areas, 40.6% of whom live in poverty, meaning they suffer of one or more social deficiencies. This means that two out of every three poor people live in urban areas [6]. Accelerated, unplanned and unsustainable urbanization also has an important impact on health [7].

In this regard, the National Institute of Public Health of Mexico points out that in marginalized urban areas, children are vulnerable to malnutrition and addictions, intrauterine growth retardation, low birth weight and decreased neurodevelopment in children as a result of the poor living conditions, inadequate nutrition and restricted access to health services [8,9,10]. However, additional environmental threats must be considered in any strategy that aims to correct health inequities, including exposure to toxic chemicals. Our group has reported about the exposure to indoor smoke [11], lead-glazed ceramics [12], arsenic [13], electronic waste [14], volatile organic compounds (VOCs) and polycyclic aromatic hydrocarbons (PAHs) in Mexican population [11]. The conclusion from these studies was that the population is permanently exposed to chemical mixtures.

In this context, children were identified as a group that was vulnerable to the effects of toxic substances because of their physical, cognitive, and physiological immaturity [15]. Even some children in urban areas in Mexico that live in vulnerable conditions and are exposed to toxic substances. Considering the data set forth above, the goal of this study was to assess the concentration levels mixture in Mexican children from marginalized urban communities.

## Methods

### Study Population

Sampling sites were selected based on the previous knowledge of the activities in each area and the availability of basic services (water, light, health services etc). Four sites were studied: i) Bellas Lomas in San Luis Potosi (BEL), a site with vehicular traffic and workshops; ii) Tercera Chica in San Luis Potosi (TC), a site with brick kilns; iii) Rincon de San Jose in San Luis Potosi (SJR), a community on the outskirts, where a hazardous waste landfill is located; and iv) Morales in San Luis Potosi (MOR) a metallurgical zone with a copper-arsenic (recently closed) and electrolytic zinc smelters. The study was conducted from 2010 to 2012, and analytical measurements were completed in late 2012. Children were randomly selected from schools located in these communities and personally interviewed to verify their eligibility to participate in the study. The inclusion criteria for children who participated were as follows: i) informed, voluntary and signed consent by the child’s parents; ii) a minimum residency period of 2 years; iii) aged between 6 and 12 years old. The research methodology was carried out with the approval of the Bioethics Committee of the Medicine School from the Autonomous University of San Luis Potosi.

### Blood and urine sample collection

First morning urine was collected in sealable plastic bottles and stored in a deep freezer until analysis (–20ºC). The extraction of the blood samples (6 mL) was performed by venipuncture with vacuum blood collection tubes (Vacutainer tubes) with EDTA as anticoagulant, the samples were stored at 4°C.

### Determination of 1-OHP in urine

1-hidroxypyrene (1-OHP) has been used as a representative biomarker of exposure in populations exposed to mixtures of PAHs [16]. 1-OHP was quantified using High Performance Liquid Chromatography (HPLC; HP1100, Agilent Technologies) using fluorescence detector (G1321 A). The limit of detection (LOD) was 1.0 nmol/L. Quality control was carried out using the standard IRIS ClinCal Recipes (Munich, Germany) 50013, 8867, and 50014. The recovery was 99%. Finally, the levels of 1-OHP in urine were adjusted by urinary creatinine (cr). Urinary creatinine was determined by using the Jaffe colorimetric method.

### Urinary trans, trans-muconic acid determination

Urinary trans, trans-muconic acid (t,t-MA) has been used as an exposure biomarker to monitoring benzene exposure. t,t-MA was quantified using HPLC (HP1100, Agilent Technologies) with a UV-Vis detector (G1314 A). The LOD was 0.03 mg/L. Control quality was verified using standard IRIS ClinCal Recipe 9969 (Munich, Germany), and the recovery rate was 97%.

### Determination of arsenic in urine

For the quantification of total arsenic (As) an aliquot of urine was treated with an acidic digestion using atomic fluorescent spectrophotometry with hydride generation (PS Analytical 10.055 Millennium Excalibur System, Deerfield Beach, FL) equipped with an empty cathode lamp. The LOD was 1μg/L. Control quality was verified using the standard ClinCheck-Urine control level I (41 ± 10 μg/L; Munich, Germany), and the recovery rate was 95%.

### Determination of manganese in urine

For determination of Manganese (Mn) levels, an aliquot of urine was treated with nitric and perchloric acid with temperature for the digestion of the sample. The quantification was performed in a Perkin-Elmer 3110 atomic absorption spectrophotometer with graphite furnace (HGA 600). The LOD was 1μg/L. Control quality was verified using the standard ClinCal-Urine level I (24.6 μg/L; Munich, Germany), and the recovery rate was 93%.

### Determination of fluoride in urine

Fluoride (F^–^) was quantified in solution using a potentiometric method with a selective ion electrode. The LOD was 0.05 mg/L. Control quality was verified using the standard ClinCheck-Urine control level I (3.8 mg/L; Munich, Germany), and the recovery rate was 96%.

### Determination of blood lead levels

The quantification of lead in blood (PbB) was performed using a Perkin-Elmer 3110 atomic absorption spectrophotometer with graphite furnace. The LOD was 1.0 μg/dL and the accuracy was 99 ± 9.0%.

### Statistics

The levels of all contaminants were compared between communities using the Kruskal–Wallis test, followed by the Dunn’s test posthoc to compare each contaminant between communities. For all statistical analyses, we used GraphPad Software version 5.0 (CA, USA). P < 0.05 was considered statistically significant.

## Results

The children anthropometric measures are shown in Table 1. According to the WHO child growth standards [17], all communities showed children with malnutrition based on their weight for age score (W/A Z scores ±2.0). According to WHO reference curves, BEL was the community with the 13.7% of chronic undernutrition followed by TC (10%), SJR (5.2%) and MOR (0%). Acute undernutrition based on weight for age was observed in all the communities (W/A z-scores <–2.0); the prevalence in SJR, MOR, BEL and TC, was 5.2, 3.7, 3.4 and 2.5%, respectively.

**Table 1:** Descriptive characteristics of participants.

Characteristics	BEL	SJR	TC	MOR

**Children (N)**	29	19	40	27
**Male (%)**	55.2	37	53	63
**Female (%)**	44.8	63	47	37
**Age (years)**	7.1 ± 1.0	7.5 ± 2.1	5.9 ± 1.6	7.6 ± 1.1
**Height (cm)**	122.7 ± 8.9	125 ± 13.1	114.5 ± 8.3	124.3 ± 9.0
**Weight (Kg)**	25.3 ± 5.4	26.1 ± 7.3	20.7 ± 5.2	25.8 ± 5.1
**BMI (Kg/m^2^)**	16.6 ± 2.2	16.4 ± 2.1	16.6 ± 2.7	16.6 ± 1.6
**Index Margination**	High	High	Very high	Medium
**Chronic Undernutrition**				
**Height for Age (H/A) (%)**^+^	3.4	5.2	2.5	3.7
**Weight for Age (W/A) (%)**^+^	13.7	5.2	10	0
**Risk activities (%)**				
**Smoking**	40	90	25	15
**Firewood Use**	0	95	90	0
**Insecticide Use**	45	50	55	40
**Well Water Use (Drinking)**	30	80	80	0
**Well Water Use (Cooking)**	72	85	95	0
**Use of Ceramic Glazed Cookware**	0	58	40	0
**Garbage Burning**	85	100	68	24

^+^ Calculated based on the Z ranges of child growth standards proposed by the World Health Organization (WHO) [17]. ^*^ Summary measure to differentiate states and municipalities according to the shortcomings that suffers population.

The levels of inorganic elements are summarized in Table 2. All median concentrations of the communities study are lower than reference values for Mn (8 μg/L) [18] but all the communities showed similar concentrations (4.46–.3 µg/L). However, MOR had the highest percentage of children with detectable Mn (72.4%) followed by SJR, BEL and TC with 52.6, 41 and 27.5%, respectively. Levels of As were lower than reference value (15 µg/L) [19] in all the communities. However, MOR had the greatest levels of As (11.0 µg/L), also had the highest percentage of children with detectable As (79.3%) followed by TC, BEL and SJR (25%, 24% and 15.7% respectively). For F^–^, all the communities had a higher median concentration in comparison to the reference value (1.5 mg/L) [20], showing median concentration similar between the communities. Also, MOR, SJR, TC and BEL, had some of children with detectable F^–^ showing percentages above 80%, 89.4%, 97.5% and 100%, respectively. Regarding to PbB, TC had lower levels than BEL and MOR (4.2, 6.4 and 6.8 µg/dL, respectively); in these communities, all participants showed detectable concentration for this compound, except SJR that showed non-detectable levels. Furthermore, MOR showed the greatest percentage of children with higher levels than the reference value of 5 µg/dL [21] (77.7%), followed by BEL and TC (65.5 and 47.5%, respectively).

**Table 2:** Assessment of exposure to metals in Mexican children urban communities.

Compound	Site	N	% Detectable	Median	Min	Max	% > RfV	RfV

Manganese*	BEL	29	**41**	4.4	2.7	23.6	25.0	8 [18]
	TC	40	**27.5**	5.8	3.5	37.1	45.4	
	SJR	19	**52.6**	5.2	2.7	17.7	33.3	
	MOR	27	**72.4**	6.3	3.1	18.7	39.1
Arsenic*	BEL	29	**24**	0.5	0.5	43.9	4.0	15 [19]
	TC	40	**25**	0.5	0.5	23.5	7.5	
	SJR	19	**15.7**	0.5	0.5	66.3	5.2	
	MOR	27	**79.3**	11.0	0.5	52.6	25.9	
**Fluoride**^$^	BEL	29	**100**	2.3	0.34	5.4	51.7
	TC	40	**97.5**	1.5	0.34	4.4	51.2	1.5 [20]
	SJR	19	**89.4**	1.2	0.34	2.4	16.0	
	MOR	27	**80**	1.7	0.34	3.5	0	
**Lead**^&^	BEL	29	**100**	6.4	2.2	22.6	65.5	
	TC	40	**100**	4.2	0.7	17.7	47.5	5 [21]
	MOR	27	**100**	6.8	2.6	19.4	77.7	

Median, Minimum and Maximum only are shown with elements with levels above detectable. *The unit is expressed as: µg/L, ^$^ mg/L and ^&^ µg/L. RfV: Reference value. ^+^statistically significant difference statiscal (p < 0.05).

On the other hand, all the children from BEL, TC and SJR had detectable urinary concentrations of 1-OHP and only MOR had 93.1% of children with detectable concentrations of this compound. Furthermore, we observed that TC showed the highest percentage of children with 1-OHP urinary levels above the reference value of 0.24 μmol/mol cr (45.0%), followed by SJR and BEL (36.8 and 24.1, respectively); children in MOR did not show concentrations above the reference values (Table 3). Additionally, we observed that TC had the highest 1-OHP exposure (0.23 µmol/mol cr) when compared with SJR, BEL and MOR (0.09, 0.06 and 0.03 µmol/mol cr, p < 0.05). For t,t-MA, SJR had the highest percentage of children with levels above the reference value of 500 µg/g cr (47.3%), followed by TC, MOR and BEL (41.0, 29.6 and 7%, respectively). Also, it was observed that the exposure levels of t,t-MA were 429.7, 427.4, 220.6 and 258.6 µg/g cr for TC, SJR, BEL and MOR, respectively.

**Table 3:** Assessment of exposure to Volatile Organic Compound in Mexican children from urban communities.

Compounds	Site	N	% Detectable	Median	Min	Max	% > RfV	RfV

1-OHP*	BEL	29	100	0.06	0.02	4.92	24.1	0.24 [16]
	TC	40	100	0.23	0.02	1.98	45.0	
	SJR	19	100	0.09	0.02	4.7	36.8	
	MOR	27	93.1	0.03	0.02	0.19	0	
*t,t*-MA**	BEL	29	100	220.6	94.3	816.5	7.0	500 [41]
	TC	40	97.5	429.7	56.3	12845	41.0	
	SJR	19	100	427.4	13.8	4994	47.3	
	MOR	27	93.1	258.6	55.6	4859	29.6	

1-OHP as an exposure biomarker to PAHs, * units: µmol/mol creatinine; t,t-MA as an exposure biomarker to benzene, ** units: µg/g creatinine. RfV: Reference value. ^+^ statistically significant difference (p < 0.05).

## Discussion

Urbanization is often understood to be a precondition for development. Cities, with their economic development, industries and services, spearhead the economic growth of any nation. However, these opportunities are not equally accessible to all. Although it is known that the inhabitants of the cities enjoy better health than the rural populations, little is known about the differences of health within the urban cities. In this sense, some research has revealed that, in urban cities, there are inequalities in health which creates a greater risk among the inner-city population of suffering from different diseases and health problems [22]. Around one-third of the world’s population live in slum conditions (828 million people) and the majority of these are located in cites of developing countries [23].

In recent years, there has been a growing interest in the intraurban scale due to the fact that some studies have shown that this population is exposed to adverse impacts of mixture of pollutants because resulting from various economic activities and high density of population of modern cities. In addition, most cases of health adverse effects in populations are caused by economic and environmental conditions such high exposure to health risk factors, poor access to health care services, and chronic malnutrition [24].

This study found that all children from the urban communities are in normal condition of nutrition according to the WHO child growth [17]. In Mexico, the short stature in preschool has been a clear decline, dropping from 26.9% in 1988 to 13.6% in 2012, down 13.3 percentage points at the national level according to the National Health and Nutrition Survey [25]. In this context, in the study areas, the child populations were in a state of adequate nutrition, this is likely due to adequate availability of food, health care, education, and health infrastructure. These factors, in turn, may be influenced by an equal distribution of resources, services, wealth, and opportunity and reflect the low marginalization index in these urban communities. This is important because of the risk for diseases and child development [26].

Alternatively, it has been observed that different environmental contaminants such as those evaluated in this study (Mn, As, F^–^, Pb, PAHs, and benzene) can affect child development and increase the risk of certain diseases. Children normally present trends in environmental exposure more accurately than adults because children are not directly exposed to occupational pollution [27]. Additionally, it has been well-established that children are potentially at more risk than adults to adverse health effects due the exposure to many environmental chemicals [28]. However, the information on human exposure to chemical mixtures is very limited, and the information in urban children is even more scarce. Thus, we assessed exposure in children in four urban communities. Mn and As levels in urine were similar between all the communities and below of the reference values (8 µg/L and 15 µg/L, respectively). Low levels of Mn, are not toxic but some studies have proposed that may produce adverse health effects at higher levels. For example, at higher values than the reference (8 μg/L) [18] has been reported neurotoxic effects [29]. About As, different studies have demonstrated that its presence has been associated with different diseases such as skin lesions, skin cancer [30], neurological, respiratory and cardiovascular diseases [31]. Moreover, it is important to highlight that the sources of As contamination include natural deposits as well as anthropogenic sources such as mining and electronics manufacturing processes and metal smelting [32]. Regarding Mn, it is a natural component in soil but the population can be exposed to this compound due to anthropogenic activities such mining [18]. For F^–^, BEL has higher values than MOR, TC and SJR; this is explained by the fact that some people from this community still cook and drink tap water, which is the main source of exposure to this element [13]. However, there have been different risk communication programs to reduce the exposure in this community [33]. These intervention programs are important because some data suggest a reduction in the intelligence quotient (IQ) score in children living in endemic fluorosis areas [34]. For PbB, TC has lower values than BEL and MOR, this is due to the fact that in this place a risk reduction program was implemented [12,13]. With respect to BEL and MOR, levels above the established values (5 µg/dL) were found. This can be explained by the use of glazed clay for cooking, as well as a smelter located 1.5 km from both communities where children are exposed to high levels of Pb [35]. The chronic exposure to Pb may be associated with neurocognitive, neurobehavioral, functional alterations [36].

On the other hand, the exposure to PAHs was assessed through the analysis of urinary 1-OHP. Jongeneelen [16] proposed a three-risk level guideline for occupational exposure to PAHs that includes urinary 1-OHP levels. Following this guideline, the first risk level or reference value that is the 95^th^ percentile in non-occupational exposed controls was set as 0.24 μmol/mol cr and 0.76 μmol/mol cr for non-smokers and smokers, respectively. The second risk level at which no biological effects are observed, urinary levels of 1-OHP for exposed workers were fixed at 1.4 μmol/mol cr (lowest reported level at which no genotoxic effects were found). Finally, two reference values were proposed as occupational exposure limits for two types of industry: 2.3 μmol/mol cr for coke ovens and 4.9 μmol/mol cr for primary aluminum production (third risk level). Interestingly, the median levels found in all the community were lower than the reference value (0.24 µmol/mol cr [16]; only TC has levels near to the reference values and this may be due to fact that this area is surrounded by brickyards [37]. Moreover, our data is comparable to that of similar studies. For example, with a study of children in Mongolia who lived near heavy traffic, demonstrated mean 1-OHP levels of 0.3 μmol/mol cr [38]; and the NHANES IV study from the USA, showed that in children aged 6–11 years demonstrate levels approximately 0.05 μmol/mol cr [39]; only levels in MOR community are lower than this value. It is important to reduce the exposure because several studies have demonstrated an association between chronic PAH exposure and incidence of lung cancer and DNA [40].

Considering exposure to benzene, the urinary levels for t,t-MA in children from this study were lower compared with the biological exposure index (BEI^®^) tt-MA guidance value of 500 μg/g cr proposed by the ACGIH [41]. which is the concentration below, nearly all people exposed occupationally should not experience adverse health effects. We found that 47.3, 41.0. 29.6 and 7% of the children from the SJR, TC, MOR and BEL community had levels above this BEI^®^ value, respectively (Table 3). It is important to reduce the exposure to benzene in children in these communities because this compound has been associated with acute myeloid leukemia [42] and is potentially associated with an increased risk of developing chronic and acute lymphoblastic leukemia in adults [43].

## Conclusions

This study measures the levels of pollutants prior to an environmental intervention, and it indicates the need for action due to exposure levels that are higher than the respective guidance values. It is possible to achieve significant reductions with a combination of factors, such as intense education, removal of all possible sources of pollutants from the environment, and effective monitoring of the affected children.

Considering the proportion of children living in urban areas in Mexico, it is important to understand that to design effective intervention programs, the exposure pathways for the children (particle inhalation, soil/dust ingestion, occupational exposure, etc.) must be identified. These programs are urgent because a greater awareness of the public health concerns associated with these exposures is needed.
